# Fibrosis or steatosis: which is the best screening target? Comment on the Brazilian evidence-based guideline for screening, diagnosis, treatment, and follow-up of metabolic dysfunction-associated steatotic liver disease (MASLD) in adult individuals with overweight or obesity

**DOI:** 10.20945/2359-4292-2024-0014

**Published:** 2024-06-03

**Authors:** Mateus Dornelles Severo

**Affiliations:** 1 Hospital Universitário de Santa Maria Santa Maria RS Brasil Hospital Universitário de Santa Maria, Santa Maria, RS, Brasil

Screening strategies are interesting for highly prevalent diseases with a detectable preclinical phase, beneficial therapeutic options, and accurate and cost-efficient screening tests. However, the screening strategy proposed in the "Brazilian evidence-based guideline for screening, diagnosis, treatment, and follow-up of metabolic dysfunction-associated steatotic liver disease (MASLD) in adult individuals with overweight or obesity" is quite problematic, as it focuses primarily on detecting steatosis (1).

The sensitivity of abdominal ultrasound in detecting moderate to severe hepatic steatosis is only 84.5% (95% confidence interval [CI] 79.5%-88.9%), which is insufficient to consider this a good screening test (2). Furthermore, both vibration-controlled transient elastography with controlled attenuation parameter and magnetic resonance imaging with proton density fat fraction, known for their higher sensitivity, are rarely available and have a high cost.

If screening for steatosis is performed before calculation of the Fibrosis-4 (FIB-4) index, many individuals at risk for advanced fibrosis (stage F2 or greater) may not be identified, especially those who are more obese, in whom ultrasound sensitivity is reduced (*i.e.*, 49% for individuals with body mass index ≥ 35 kg/m^2^) (3). Therefore, the opportunity to offer better evaluation, treatment, and follow-up for these patients without documented steatosis will be lost.

The pretest probability for detecting steatosis with FIB-4 calculation among overweight and obese individuals is, respectively, 69.99% (95% CI 65.40%-74.21%) and 75.27% (95% CI 70.90%-79.18%). These rates are sufficiently high to justify screening directly through the FIB-4 calculation (4).

Screening strategies focused on detecting increased risk of advanced fibrosis (F2 or greater) are much cheaper and simpler to implement than those recommended in the guideline. They also categorized the patients into two groups:

- FIB-4 < 1.3: These patients require routine follow-ups as per overweight and obesity management guidelines.
- FIB-4 ≥ 1.3: These patients require further evaluation with more complex tests to confirm the presence of advanced fibrosis.

The projected prevalence rates of overweight and obesity in Brazil by 2030 are 68.1% and 29.6%, respectively (5). Thus, the screening strategy proposed in the Brazilian guideline (1) lacks accuracy and economic viability and is unable to serve the Brazilian population, deserving a review.
